# Sex-specific differences in the salivary microbiome of caries-active children

**DOI:** 10.1080/20002297.2019.1653124

**Published:** 2019-08-28

**Authors:** Stephanie Ortiz, Elisa Herrman, Claudia Lyashenko, Anne Purcell, Kareem Raslan, Brandon Khor, Michael Snow, Anna Forsyth, Dongseok Choi, Tom Maier, Curtis A. Machida

**Affiliations:** a Academic DMD Program, Oregon Health & Science University School of Dentistry, Portland, OR, US; b Department of Integrative Biomedical and Diagnostic Sciences, Oregon Health & Science University School of Dentistry, Portland, OR, US; c Department of Pediatric Dentistry, Oregon Health & Science University School of Dentistry, Portland, OR, US; d OHSU-PSU School of Public Health, Kyunghee University, Portland, OR, US; e Graduate School of Dentistry, Kyunghee University, Seoul, South Korea

**Keywords:** Salivary microbiome, sex differences, dental caries, children, oral microbiome

## Abstract

**Background and Objectives**: Dental caries is a chronic disease affecting young children and has multi-factorial risk factors. The purpose of this work was to identify sex-specific differences in the salivary microbiota within caries-active children.

**Design**: Saliva specimens were collected from 85 children (boys: 41; girls: 44) between the ages of 2-12 years. Salivary microbial DNA was subjected to PCR amplification using V3-V4 16S rDNA-specific primers and next-generation sequencing.

**Results**: Significant sex differences in salivary microbiota were found between caries-active boys versus caries-active girls. *Neisseria flavescens, Rothia aeria*, and *Haemophilus pittmaniae* were found at significantly higher levels in caries-active boys. In contrast, *Lactococcus lactis*, *Selenomonas* species HOT 126, *Actinobaculum* species HOT 183, *Veillonella parvula*, and *Alloprevotella* species HOT 473 were found at significantly higher levels in caries-active girls.

**Conclusion**: We have found the acid-generating, *cariogenic Lactococcus lactis* to be much more abundant in caries-active girls than caries-active boys, indicating that this microorganism may play a more significant role in shaping the cariogenic microbiome in girls. In addition, in caries-active girls, *Alloprevotella* species HOT 473 was the only species that exhibited both significant sex differences (4.4-fold difference; p=0.0003) as well as high abundance in numbers (1.85% of the total microbial population).

Dental caries is a common and undertreated chronic pediatric disease, exhibiting complex multifactorial etiology, lasting health effects, and serious socio-economic consequences [1–3]. The development and progression of caries are impacted by several socio-demographic risk factors, including lower education and poor oral hygiene and dietary behaviors, as well as microbiological and genetic components, including dysbiosis of the oral microbiota, altered saliva composition and flow rate, and genetic variability [1–3].

Continued cycling of enamel demineralization and remineralization is the biological basis of the caries process, with acid production by cariogenic bacteria resulting in demineralization of tooth structure, and buffering actions of saliva and fluoride promoting remineralization. An imbalance in these two processes results in caries progression and disease.

A balanced oral microbiome is a foundational element of oral health; dysbiosis and the proliferation of cariogenic microbial species result in increased tooth demineralization and cavitation [4]. Microbial species considered classically cariogenic include *Streptococcus mutans* and *Lactobacillus* [4,5]. Additional microorganisms that are associated with caries development include *Scardovia wiggsiae, Firmicutes, Veillonella* HOT 780, *Slackia exigua, Porphyromonas, Granulicatella elegans*, and *Actinomyces* [4–7]. These cariogenic bacteria produce an acidic environment by breaking down fermentable carbohydrates, and support the premise that diet and specific nutrients are key determinants in dental caries. The frequency of carbohydrate consumption will influence or determine the duration of acidic conditions within the oral cavity.

Recent studies demonstrate widely observed sex differences in caries prevalence, with females at higher risk to experience caries than males [8,9]. The sex gap in caries experience is reported to increase progressively with age [10]. In children, males and females express nearly equal caries rates – a pattern that shifts by the time of adolescence to females expressing higher caries susceptibility [10,11].

The higher susceptibility of females to dental caries may be due to several factors, including genetic variations, dietary factors, hormonal fluctuations, salivary differences, and social factors, all of which influence the composition of the oral microbiome [12,13]. Studies have shown differences between the sexes with lower salivary flow rates in women, in addition to lower pH and IgA levels, potentially leading to higher caries incidence [12,14,15]. Genetic variations in factors influencing taste, enamel proteins, and enamel structure are also linked to this trend [12,13,16]. Regarding patterns of tooth eruption, female teeth generally erupt at an earlier age, exposing teeth to a potential caries-inducing environment for longer durations of time [12]. Caries rates in women increase progressively throughout lifetimes, particularly during the reproductive years. Several physiological changes occur during pregnancy including hormonal fluctuations, immune suppression, dietary changes, and salivary flow [12,13]. Social factors that potentially help determine sex differences in caries experience include lower literacy rates in women globally, responsibility for food preparation, experience of domestic violence, and higher rates of eating disorders [12].

In this paper, we identified sex-specific differences in the salivary microbiome in caries-active children and describe the cariogenic salivary microbiome to gain further understanding of caries progression in females that may account for the observed global sex dissimilarity in caries experience.

## Materials and methods

### Participant selection and demographics

We selected study participants primarily from patients visiting the OHSU Pediatric Dental Clinic at Doernbecher Children’s Hospital, or at the OHSU School of Dentistry at the Robertson Life Sciences Building on the South Waterfront Campus. Some caries-free patients were obtained from an external practitioner’s dental clinic operated in Albany, Oregon. The study protocol was reviewed and approved by the OHSU Institutional Review Board (IRB; protocol #6535), and permissions and consent to participate were obtained from the parents or guardians of all children selected for this study. For older children, age 7 years or more, child assent and permissions were also obtained. This study is part of a larger study that examines the efficacy and use of adjunctive clinical therapies in caries-active children, and involves the use of specimens obtained before the application of any adjunctive therapies or other procedures that were applied as part of routine dental care. The inclusion parameters for study participation were children ages 2–12 years, in good general health (ASA I or II). The ASA physical status classification system is used by the American Association of Anesthesiologists to assess the fitness of patients before surgery; ASA I and ASA II designate a healthy individual, or an individual with mild systemic disease, respectively. Children excluded from the study were those who had undergone antibiotic treatment within the previous three month-period or were undergoing current orthodontic therapies, as well as children with recent exposure to topical fluoride or antiseptic mouth rinses. Eighty-five children were enrolled in this study (41 boys and 44 girls). Of the 85 children, 64 children were caries-active and exhibited DMFT scores of 1–15 at the time of appointment (DMFT average: 8.6) and 21 children were caries-free individuals, who demonstrated no apparent evidence of carious lesions. The numbers of caries-active boys, caries-active girls, caries-free boys, and caries-free girls were 28, 33, 13, and 11 individuals, respectively. Caries status and prevalence were determined by clinical examination, both visual and tactile, and with the use of the decay-missing-filled (DMFT) index and confirmatory radiographs. For children, 5 years of age or younger, tooth surfaces were examined to assess caries status.

### Saliva collection, DNA extraction, and human oral microbial identification

Non-stimulated saliva specimens (1–2 ml) were collected from the oral cavity of enrolled participants using the OMNIgene-ORAL collection and stabilization kit for microbial DNA (DNA Genotek; OM-501). In some cases, especially for young children, sterile bulbs or sterile swabs were used to collect saliva from the oral cavity, prior to placement of saliva within the OMNIgene-ORAL stabilization reagent. OMNIgene-ORAL stabilizes samples at the point of collection until transport to the laboratory, and permits long-term storage up to 1 year under ambient temperatures. The OHSU Gene Profiling Shared Resources Laboratory extracted microbial genomic DNA from the saliva specimens, using kits compatible with the OMNIgene-ORAL stabilization reagent, and subsequently quantified purified DNA by absorbance at 260 nm. Microbial genomic DNA was then subjected to agarose gel electrophoresis, in the presence of ethidium bromide, and nucleic acid integrity was then documented using the Bio-Rad Gel Doc EZ Gel Documentation system (product 1,708,270; Bio-Rad Laboratories, Hercules, CA). Microbial DNA was then subjected to PCR amplification using V3-V4 16S rDNA-specific primers and next-generation sequencing with high-throughput Illumina sequencing in the Human Oral Microbial Identification *Next Generation Sequencing (NGS)* HOMI*NGS* Laboratory at the Forsyth Institute (Cambridge, MA). HOMI*NGS* as defined by the Forsyth Institute ‘permit species-level identification of nearly 600 oral bacterial taxa and genus-level identification of remaining sequences for 129 genera’. This methodology has been pioneered by Dr. Bruce J Paster, has been used extensively in microbiome studies, including oral microbiome analyses, and is described in representative publications [17–19]. Briefly, oral taxa identification and abundance of microorganisms were assessed with the ProbeSeq program, where sequence targets were matched initially with species probes and then sequentially with genus probes. Sequence targets not uniquely matched to either species or genus probes were then assessed as unmatched.

### Statistical analyses

The relative abundance of oral microorganisms was compared according to caries status and by sex. All non-zero NGS count data were included in computing the relative abundance within each participant, and then averaged by the group. The differential abundance analyses of NGS count data of oral microorganisms were performed by a zero-inflated negative binomial regression model by the zinbwave and edgeR packages in the R statistical language (http://www.r-project.org) [20,21]. The ZINB-WaVE method implemented the zinbwave package and efficiently identified excess zero count data and provided microorganism and sample-specific weights. These weights were then incorporated into the dispersion estimates of the negative binomial regression model in edgeR, in order to determine or recover the power of statistical tests of relative abundance of microorganisms between caries status or by sex. Only 298 microorganisms detected in at least nine participants were kept in the analysis to stabilize dispersion parameter estimates. Two-sided p-values for testing relative abundance by caries status or by sex were adjusted by the false discovery rate (FDR) for multiple test correction [22]. Fold changes larger than 1.5, with the FDR adjusted p-value of < 0.05 were considered statistically significant [22].

## Results

### Abundance of salivary microorganisms within caries-active boys versus caries-active girls

**Figure 1.**
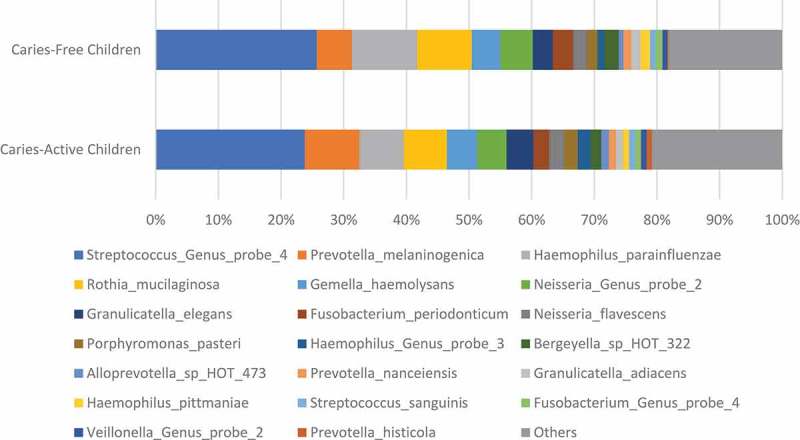
Abundance of top 20 salivary microorganisms found in caries-active and caries-free children. Plots were calculated based on select information displayed in Table 1.

**Figure 2.**
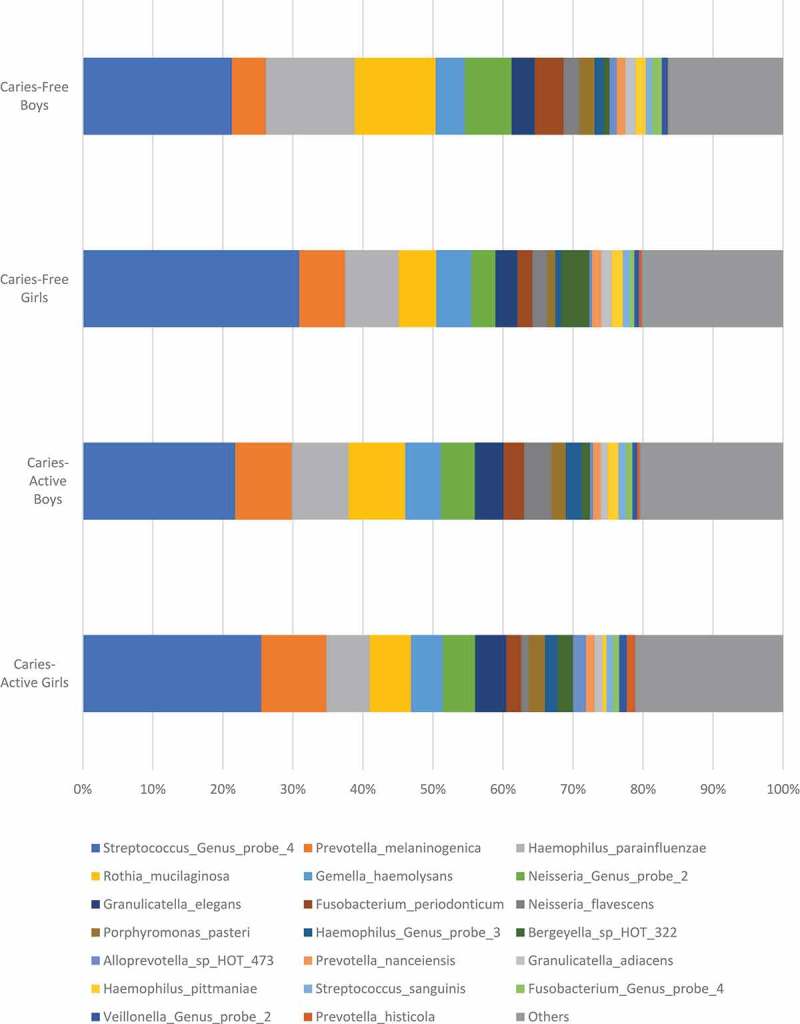
Abundance of top 20 salivary microorganisms found in caries-active and caries-free children differentiated by sex. Plots were calculated based on select information displayed in Table 1.

Table 1 ranks the abundance of microorganisms in caries-active girls, caries-active boys, caries-free girls, caries-free boys, caries-active children, and caries-free children; using box representations, Figures 1 and 2 display the abundance of the top 20 microorganisms differentiated by caries status and sex, respectively. Tables 2 (caries-active boys versus caries-active girls) and 3 (caries-free boys versus caries-free girls) compares the fold changes for each microorganism between boys versus girls and ranks the fold changes with high positive numbers representing higher proportional representation of microorganisms in boys and extremely low negative values representing higher proportional representation of microorganisms in girls. The abundance list in Table 1 is sorted according to rankings of microorganisms observed in caries-active children and then applied in the same sequence for all remaining groups, and lists only the top 100 microorganisms. In Tables 2 and 3, microorganisms found in significantly-higher proportions in boys versus girls are shaded in gray, and in general, only lists microorganisms with statistically significant differences according to sex. In Table 1, *Streptococci* are the most abundant microorganisms observed in all groups. *Prevotella melaninogenica* is the second most abundant microorganism in caries-active children while it is the fourth most abundant in caries-free children. In caries-active boys, *Neisseria flavescens* and *Haemophilus pittmaniae* were the only two microorganisms (shaded in gray in Table 1) that exhibited both significant sex differences (> 1.5-fold changes with FDR adjusted p < 0.05) and abundance comprising > 0.5% of the microbial population (Tables 1 and 2). *N. flavescens* and *H. pittmaniae* were abundant in caries-active boys, comprising 3.92% and 1.53%, respectively, of the salivary microbial population. In caries-active girls, *Alloprevotella* species HOT 473 was the only species that exhibited significant sex differences (4.4-fold difference between caries-active girls and caries-active boys; p = 0.0003) and high abundance in numbers (1.85% of the total microbial population in caries-active girls).

**Table 1.** Abundance of salivary microorganisms found in caries-active and caries-free children.

Probe ID	Microorganism	Caries-Active Girls	Caries-Active Boys	Caries-Free Girls	Caries-Free Boys	Caries-Active Children	Caries-Free Children
GP-081	Streptococcus_Genus_probe_4	25.49%	21.76%	30.93%	21.28%	23.78%	25.70%
PR-14	Prevotella_melaninogenica	9.26%	8.06%	6.50%	4.84%	8.71%	5.60%
HA-05	Haemophilus_parainfluenzae	6.21%	8.13%	7.69%	12.72%	7.09%	10.42%
RO-03	Rothia_mucilaginosa	5.89%	8.08%	5.36%	11.55%	6.90%	8.71%
GE-02	Gemella_haemolysans	4.56%	5.07%	5.02%	4.16%	4.79%	4.56%
GP-060	Neisseria_Genus_probe_2	4.60%	4.87%	3.43%	6.68%	4.72%	5.19%
GR-02	Granulicatella_elegans	4.47%	4.12%	3.16%	3.29%	4.31%	3.23%
FU-10	Fusobacterium_periodonticum	2.09%	2.91%	2.11%	4.10%	2.47%	3.18%
NE-03	Neisseria_flavescens	1.10%	3.92%	2.15%	2.26%	2.39%	2.21%
PO-09	Porphyromonas_pasteri	2.31%	2.05%	1.14%	2.18%	2.19%	1.70%
GP-040	Haemophilus_Genus_probe_3	1.81%	2.28%	0.82%	1.31%	2.02%	1.09%
BE-02	Bergeyella_sp_HOT_322	2.20%	1.18%	4.05%	0.84%	1.74%	2.31%
AL-04	Alloprevotella_sp_HOT_473	1.85%	0.43%	0.40%	1.05%	1.20%	0.75%
PR-79	Prevotella_nanceiensis	1.19%	1.10%	1.30%	1.20%	1.15%	1.25%
GR-01	Granulicatella_adiacens	1.17%	1.00%	1.48%	1.49%	1.09%	1.49%
HA-07	Haemophilus_pittmaniae	0.57%	1.53%	1.61%	1.43%	1.01%	1.51%
ST-20	Streptococcus_sanguinis	0.93%	1.07%	0.99%	0.88%	1.00%	0.94%
GP-038	Fusobacterium_Genus_probe_4	0.89%	0.93%	0.63%	1.40%	0.91%	1.05%
GP-089	Veillonella_Genus_probe_2	1.08%	0.68%	0.67%	0.83%	0.90%	0.76%
PR-09	Prevotella_histicola	1.19%	0.35%	0.39%	0.06%	0.80%	0.21%
PO-24	Porphyromonas_pasteri	0.65%	0.78%	0.77%	0.89%	0.71%	0.84%
PR-25	Prevotella_salivae	0.86%	0.51%	0.76%	0.06%	0.70%	0.38%
GP-039	Gemella_Genus_probe	0.65%	0.67%	0.78%	0.57%	0.66%	0.66%
GP-126	Streptococcus_Genus_probe_1	0.51%	0.62%	0.45%	0.28%	0.56%	0.36%
LE-16	Leptotrichia_sp_HOT_417	0.65%	0.45%	0.26%	0.08%	0.56%	0.16%
VE-08	Veillonella_sp_HOT_780	0.67%	0.32%	0.32%	0.41%	0.51%	0.37%
LE-06	Leptotrichia_hongkongensis	0.43%	0.52%	0.40%	0.13%	0.47%	0.25%
PR-22	Prevotella_pallens	0.42%	0.50%	0.25%	0.05%	0.46%	0.14%
LE-07	Leptotrichia_shahii	0.28%	0.64%	0.39%	0.17%	0.44%	0.27%
GP-019	Campylobacter_Genus_probe_2	0.46%	0.42%	0.19%	0.20%	0.44%	0.19%
NE-19	Neisseria_subflava	0.20%	0.62%	0.53%	0.32%	0.39%	0.41%
GE-04	Gemella_sanguinis	0.33%	0.44%	0.67%	0.66%	0.38%	0.67%
VE-21	Veillonella_atypica	0.48%	0.26%	0.60%	0.07%	0.38%	0.31%
GP-073	Rothia_Genus_probe	0.32%	0.43%	0.27%	0.38%	0.37%	0.33%
RO-01	Rothia_aeria	0.20%	0.55%	0.22%	0.20%	0.36%	0.21%
GP-112	Haemophilus_Genus_probe_2	0.39%	0.32%	0.18%	0.31%	0.36%	0.25%
VE-03	Veillonella_dispar	0.49%	0.20%	0.22%	0.11%	0.36%	0.16%
AG-06	Aggregatibacter_sp_HOT_513	0.33%	0.36%	0.03%	0.09%	0.35%	0.06%
GP-099	Aggregatibacter_Genus_probe_1	0.27%	0.44%	0.18%	0.26%	0.34%	0.22%
AB-01	Abiotrophia_defectiva	0.44%	0.22%	0.19%	0.46%	0.34%	0.34%
NE-16	Neisseria_flavescens	0.03%	0.64%	0.00%	0.02%	0.31%	0.01%
VE-20	Veillonella_atypica	0.40%	0.21%	0.26%	0.04%	0.31%	0.14%
LA-29	Lautropia_mirabilis	0.26%	0.35%	0.84%	0.77%	0.30%	0.80%
CL-03	Ruminococcaceae[G-1]_sp_HOT_075	0.33%	0.25%	0.08%	0.07%	0.29%	0.07%
RO-02	Rothia_dentocariosa	0.35%	0.20%	0.31%	0.32%	0.28%	0.32%
NE-08	Neisseria_pharyngis	0.13%	0.41%	0.04%	0.07%	0.26%	0.06%
OR-01	Oribacterium_sinus	0.21%	0.28%	0.21%	0.59%	0.24%	0.42%
LE-13	Leptotrichia_sp_HOT_221	0.32%	0.12%	0.05%	0.05%	0.23%	0.05%
GP-049	Leptotrichia_Genus_probe_3	0.27%	0.16%	0.06%	0.02%	0.22%	0.04%
GP-050	Leptotrichia_Genus_probe_4	0.18%	0.27%	0.12%	0.29%	0.22%	0.21%
GE-07	Gemella_morbillorum	0.20%	0.23%	0.04%	0.21%	0.21%	0.13%
AL-11	Alloprevotella_sp_HOT_914	0.22%	0.20%	0.07%	0.19%	0.21%	0.14%
VE-07	Veillonella_rogosae	0.11%	0.33%	0.40%	0.67%	0.21%	0.54%
LE-11	Leptotrichia_sp_HOT_218	0.31%	0.09%	0.09%	0.00%	0.21%	0.04%
FU-12	Fusobacterium_nucleatum_subsp_animalis	0.11%	0.28%	0.02%	0.01%	0.18%	0.02%
PR-18	Prevotella_nigrescens	0.16%	0.21%	0.19%	0.03%	0.18%	0.10%
PR-51	Prevotella_veroralis	0.28%	0.06%	0.07%	0.01%	0.18%	0.04%
TM-05	TM7[G-1]_sp_HOT_352	0.13%	0.19%	0.17%	0.12%	0.16%	0.15%
LE-26	Leptotrichia_sp_HOT_215	0.15%	0.16%	0.13%	0.08%	0.15%	0.10%
GP-005	Alloprevotella_Genus_probe	0.20%	0.09%	0.08%	0.15%	0.15%	0.12%
GP-100	Aggregatibacter_Genus_probe_2	0.09%	0.21%	0.03%	0.08%	0.15%	0.06%
NE-18	Neisseria_oralis	0.11%	0.18%	0.17%	0.06%	0.14%	0.11%
GP-004	Actinomyces_Genus_probe_4	0.16%	0.12%	0.18%	0.13%	0.14%	0.15%
PR-26	Prevotella_scopos	0.09%	0.19%	0.06%	0.07%	0.13%	0.07%
GE-05	Gemella_morbillorum	0.10%	0.15%	0.03%	0.11%	0.12%	0.07%
TM-01	TM7[G-1]_sp_HOT_346	0.20%	0.02%	0.00%	0.07%	0.12%	0.04%
CO-03	Corynebacterium_matruchotii	0.13%	0.09%	0.54%	0.20%	0.11%	0.36%
EU-03	Peptostreptococcaceae[XI][G-1][Eubacterium]_sulci	0.09%	0.14%	0.07%	0.04%	0.11%	0.05%
ME-01	Megasphaera_micronuciformis	0.17%	0.03%	0.13%	0.01%	0.11%	0.06%
PR-70	Prevotella_pallens	0.14%	0.07%	0.04%	0.03%	0.11%	0.03%
LE-22	Leptotrichia_wadei	0.13%	0.08%	0.12%	0.00%	0.11%	0.06%
VE-06	Veillonella_parvula	0.18%	0.02%	0.02%	0.01%	0.11%	0.02%
LA-28	Lactococcus_lactis	0.19%	0.00%	0.00%	0.00%	0.10%	0.00%
LA-06	Lachnoanaerobaculum_umeaense	0.11%	0.08%	0.11%	0.16%	0.10%	0.13%
PR-20	Prevotella_oris	0.11%	0.08%	0.17%	0.02%	0.10%	0.09%
KI-03	Kingella_oralis	0.13%	0.06%	0.05%	0.02%	0.10%	0.03%
GP-067	Porphyromonas_Genus_probe_2	0.08%	0.12%	0.02%	0.12%	0.10%	0.07%
GP-118	Moraxella_Genus_probe_1	0.00%	0.22%	0.00%	0.00%	0.10%	0.00%
PR-21	Prevotella_oulorum	0.12%	0.07%	0.06%	0.01%	0.10%	0.03%
GP-069	Prevotella_Genus_probe_2	0.10%	0.09%	0.05%	0.07%	0.10%	0.06%
SO-01	Solobacterium_moorei	0.08%	0.11%	0.11%	0.07%	0.10%	0.09%
CL-04	Ruminococcaceae[G-2]_sp_HOT_085	0.11%	0.07%	0.04%	0.02%	0.09%	0.03%
GE-03	Gemella_morbillorum	0.08%	0.10%	0.02%	0.09%	0.09%	0.06%
GP-096	Fusobacterium_Genus_probe_3	0.11%	0.07%	0.04%	0.12%	0.09%	0.09%
PE-21	Peptostreptococcus_stomatis	0.08%	0.10%	0.06%	0.08%	0.09%	0.07%
PO-26	Porphyromonas_sp_HOT_930	0.09%	0.08%	0.00%	0.00%	0.09%	0.00%
AC-20	Actinomyces_sp_HOT_172	0.10%	0.07%	0.16%	0.07%	0.09%	0.11%
OR-06	Oribacterium_asaccharolyticum	0.09%	0.08%	0.06%	0.06%	0.08%	0.06%
GP-130	Veillonella_Genus_probe_1	0.11%	0.05%	0.14%	0.03%	0.08%	0.08%
PR-06	Prevotella_denticola	0.07%	0.09%	0.01%	0.00%	0.08%	0.00%
CA-37	Catonella_morbi	0.06%	0.09%	0.05%	0.13%	0.08%	0.09%
AL-09	Alloprevotella_tannerae	0.07%	0.08%	0.01%	0.01%	0.07%	0.01%
PR-39	Prevotella_sp_HOT_309	0.09%	0.06%	0.04%	0.01%	0.07%	0.03%
GP-113	Kingella_Genus_probe_1	0.07%	0.08%	0.05%	0.08%	0.07%	0.06%
PO-03	Porphyromonas_endodontalis	0.06%	0.08%	0.00%	0.00%	0.07%	0.00%
GP-076	Selenomonas_&_Centipeda_Genus_probe	0.12%	0.02%	0.21%	0.34%	0.07%	0.28%
NE-02	Neisseria_elongata	0.07%	0.07%	0.05%	0.13%	0.07%	0.09%

Microorganisms shaded in gray have been found to have significant sex differences, as demonstrated in Tables 2, 3, and 4, as well as high abundance comprising greater than 1.5% of the total microbial population.

**Table 2.** Significant differences in salivary microorganisms found in caries-active boys versus caries-active girls.

Probe ID	Microorganism	Average Counts per Million	Fold Changes	p-value	FDR p-value
GP-057	Moraxella_Genus_probe_2	339.5	171.0	0.0000	0.0004
GP-127	Streptococcus_Genus_probe_2	434.2	26.4	0.0000	0.0004
NE-16	Neisseria_flavescens	2056.0	20.7	0.0011	0.0287
RO-01	Rothia_aeria	3918.0	3.9	0.0001	0.0114
HA-07	Haemophilus_pittmaniae	15,037.3	3.8	0.0004	0.0177
AL-04	Alloprevotella_sp_HOT_473	10,366.4	−4.4	0.0003	0.0169
VE-06	Veillonella_parvula	676.9	−5.4	0.0005	0.0194
PR-41	Prevotella_sp_HOT_315	54.4	−7.3	0.0007	0.0271
LA-03	Lachnoanaerobaculum_sp_HOT_083	75.9	−8.9	0.0012	0.0295
AC-03	Actinobaculum_sp_HOT_183	266.6	−9.8	0.0010	0.0287
SE-37	Selenomonas_sp_HOT_126	209.9	−21.5	0.0009	0.0283
LA-28	Lactococcus_lactis	498.9	−92.2	0.0002	0.0169

Microorganisms exhibiting positive value fold changes (shaded in gray) indicate higher prevalence in caries-active boys compared to caries-active girls.Microorganisms exhibiting negative value fold changes (unshaded) indicate higher prevalence in caries-active girls compared to caries-active boys.

**Table 3.** Significant differences in salivary microorganisms found in caries-free boys versus caries-free girls.

Probe ID	Microorganism	Average Counts per Million	Fold Changes	p-value	FDR p-value
LA-31	Lachnospiraceae[G-3]_sp_HOT_100	527.2	39.5	0.0023	0.0341
BE-03	Bergeyella_sp_HOT_900	313.2	21.8	0.0032	0.0427
VE-21	Veillonella_atypica	3025.7	−6.2	0.0020	0.0314
BE-02	Bergeyella_sp_HOT_322	24,673.6	−6.2	0.0029	0.0412
PR-25	Prevotella_salivae	4980.9	−6.8	0.0033	0.0427
GP-095	Fusobacterium_Genus_probe_2	412.7	−7.1	0.0008	0.0173
ME-01	Megasphaera_micronuciformis	842.4	−11.5	0.0006	0.0144
PR-20	Prevotella_oris	952.2	−11.7	0.0003	0.0118
AC-36	Actinomyces_naeslundii	347.0	−16.9	0.0003	0.0118
SE-18	Selenomonas_sputigena	126.9	−26.8	0.0015	0.0263
GP-022	Cardiobacterium_Genus_probe	1195.8	−28.6	0.0001	0.0085
PR-23	Prevotella_pleuritidis	338.1	−28.7	0.0014	0.0261
LE-22	Leptotrichia_wadei	865.2	−37.4	0.0006	0.0144
ST-09	Streptococcus_anginosus	185.0	−49.3	0.0002	0.0111
AC-19	Actinomyces_sp_HOT_171	166.9	−69.4	0.0001	0.0085
LE-19	Leptotrichia_sp_HOT_498	398.4	−78.7	0.0001	0.0085
OR-08	Oribacterium_sp_HOT_078	386.3	−87.3	0.0008	0.0180
LE-11	Leptotrichia_sp_HOT_218	1106.9	−102.0	0.0019	0.0311
AG-10	Aggregatibacter_sp_HOT_949	117.6	−137.3	0.0013	0.0250
AC-18	Actinomyces_sp_HOT_170	636.4	−159.1	0.0002	0.0111
PR-19	Prevotella_oralis	172.0	−206.2	0.0004	0.0129
PR-15	Prevotella_micans	687.0	−298.6	0.0006	0.0144
SE-05	Selenomonas_noxia	2956.5	−357.0	0.0000	0.0060

Microorganisms exhibiting positive value fold changes (shaded in gray) indicate higher prevalence in caries-active boys compared to caries-active girls.  Microorganisms exhibiting negative value fold changes (unshaded) indicate higher prevalence in caries-active girls compared to caries-active boys.

**Table 4.** Ranking of salivary microorganisms found in caries-active children versus caries-free children.

Probe ID	Microorganism	Average Counts per Million	Fold Changes	p-value	FDR p-value
BA-17	Bacteroidetes[G-5]_sp_HOT_511	115.3	531.4	0.0004	0.0031
PR-23	Prevotella_pleuritidis	338.1	504.9	0.0001	0.0010
TR-02	Treponema_denticola	93.9	370.2	0.0001	0.0009
ST-21	Streptococcus_sobrinus	151.1	331.3	0.0036	0.0171
LE-19	Leptotrichia_sp_HOT_498	398.4	241.0	0.0001	0.0010
LA-28	Lactococcus_lactis	498.9	209.7	0.0144	0.0455
GP-057	Moraxella_Genus_probe_2	339.5	189.7	0.0055	0.0223
LE-11	Leptotrichia_sp_HOT_218	1106.9	187.4	0.0038	0.0178
NE-16	Neisseria_flavescens	2056.0	171.4	0.0082	0.0299
PO-26	Porphyromonas_sp_HOT_930	791.7	132.8	0.0023	0.0120
AG-01	Aggregatibacter_actinomycetemcomitans	85.8	127.9	0.0031	0.0158
GP-012	Bacteroidetes[G-5]_Genus_probe	66.3	96.5	0.0004	0.0035
PR-06	Prevotella_denticola	520.6	92.0	0.0040	0.0184
GP-127	Streptococcus_Genus_probe_2	434.2	70.3	0.0020	0.0110
EU-02	Peptostreptococcaceae[XI][G-1][Eubacterium]_infirmum	64.7	65.5	0.0007	0.0051
TR-04	Treponema_lecithinolyticum	72.3	63.4	0.0055	0.0223
CA-11	Capnocytophaga_haemolytica	73.3	56.7	0.0050	0.0214
TR-33	Treponema_sp_HOT_257	64.5	55.9	0.0045	0.0199
GP-086	Treponema_Genus_probe_4	77.0	54.2	0.0058	0.0228
MY-10	Mycoplasma_salivarium	81.8	50.6	0.0168	0.0497
LA-08	Lachnospiraceae[G-2]_sp_HOT_096	276.9	39.3	0.0088	0.0313
TR-58	Treponema_sp_HOT_237	62.1	35.1	0.0034	0.0168
LA-11	Butyrivibrio_sp_HOT_455	369.3	32.7	0.0055	0.0223
CA-28	Capnocytophaga_sp_HOT_902	73.8	32.4	0.0048	0.0207
PE-01	Peptococcus_sp_HOT_167	61.1	29.7	0.0070	0.0262
AL-09	Alloprevotella_tannerae	482.6	27.4	0.0093	0.0317
FU-12	Fusobacterium_nucleatum_subsp_animalis	1129.1	25.2	0.0006	0.0045
TR-05	Treponema_maltophilum	54.0	14.5	0.0156	0.0476
PR-51	Prevotella_veroralis	1406.4	13.8	0.0140	0.0449
AC-07	Actinomyces_gerencseriae	67.6	13.1	0.0148	0.0464
GP-049	Leptotrichia_Genus_probe_3	1238.6	8.8	0.0059	0.0229
PO-09	Porphyromonas_pasteri	26,451.6	−4.1	0.0114	0.0377
GR-02	Granulicatella_elegans	49,220.8	−4.6	0.0091	0.0316
RO-03	Rothia_mucilaginosa	99,788.6	−4.8	0.0089	0.0313
GP-038	Fusobacterium_Genus_probe_4	9703.2	−5.1	0.0008	0.0056
GP-112	Haemophilus_Genus_probe_2	4763.9	−5.3	0.0168	0.0497
HA-05	Haemophilus_parainfluenzae	101,160.9	−5.4	0.0011	0.0070
FU-10	Fusobacterium_periodonticum	29,184.7	−5.6	0.0035	0.0171
CA-04	Campylobacter_gracilis	635.3	−6.3	0.0161	0.0484
GP-050	Leptotrichia_Genus_probe_4	2282.6	−6.3	0.0030	0.0158
CA-31	Cardiobacterium_hominis	590.2	−6.6	0.0039	0.0180
GP-039	Gemella_Genus_probe	8474.1	−6.7	0.0016	0.0091
GP-025	Corynebacterium_Genus_probe	360.7	−7.2	0.0041	0.0185
PO-24	Porphyromonas_pasteri	8037.8	−7.4	0.0008	0.0053
ST-29	Stenotrophomonas_maltophilia	51.4	−7.5	0.0069	0.0259
LA-06	Lachnoanaerobaculum_umeaense	1228.0	−7.5	0.0004	0.0033
RO-02	Rothia_dentocariosa	3995.8	−7.8	0.0018	0.0101
GP-046	Lactobacillus_Genus_probe_3	74.2	−7.8	0.0047	0.0206
GR-01	Granulicatella_adiacens	14,304.5	−7.9	0.0000	0.0006
HA-07	Haemophilus_pittmaniae	15,037.3	−8.0	0.0014	0.0082
CA-30	Capnocytophaga_sputigena	766.6	−8.1	0.0005	0.0043
GP-023	Catonella_Genus_probe	354.0	−8.3	0.0085	0.0305
OR-01	Oribacterium_sinus	3558.9	−8.7	0.0003	0.0031
LE-15	Leptotrichia_sp_HOT_392	620.5	−8.7	0.0067	0.0255
CA-12	Capnocytophaga_leadbetteri	864.1	−11.3	0.0003	0.0031
GE-04	Gemella_sanguinis	5988.5	−11.7	0.0001	0.0009
GE-02	Gemella_haemolysans	75,652.7	−11.9	0.0000	0.0005
ST-09	Streptococcus_anginosus	185.0	−13.4	0.0152	0.0473
PO-27	Porphyromonas_sp_HOT_284	472.5	−13.7	0.0010	0.0063
GP-003	Actinomyces_Genus_probe_3	990.3	−13.8	0.0001	0.0015
GP-081	Streptococcus_Genus_probe_4	374,334.7	−14.1	0.0000	0.0000
GP-083	TM7_Genus_probe	789.1	−14.1	0.0001	0.0014
CA-09	Capnocytophaga_gingivalis	447.6	−15.0	0.0001	0.0015
PR-73	Prevotella_sp_HOT_317	211.0	−15.2	0.0127	0.0410
VE-07	Veillonella_rogosae	3703.2	−16.1	0.0001	0.0016
CA-32	Cardiobacterium_valvarum	116.1	−16.3	0.0010	0.0063
GP-020	Capnocytophaga_Genus_probe_2	406.4	−16.9	0.0000	0.0002
AC-24	Actinomyces_lingnae	868.9	−17.0	0.0001	0.0013
CA-01	Campylobacter_concisus	216.9	−17.3	0.0003	0.0031
LE-25	Leptotrichia_sp_HOT_223	197.4	−17.4	0.0058	0.0228
AC-30	Actinomyces_sp_HOT_877	81.4	−17.8	0.0120	0.0392
SE-05	Selenomonas_noxia	2956.5	−19.6	0.0095	0.0320
PE-28	Peptostreptococcaceae[XI][G-7]_sp_HOT_922	188.2	−20.9	0.0096	0.0320
TM-14	TM7[G-1]_sp_HOT_952	227.5	−21.1	0.0006	0.0046
CO-02	Corynebacterium_durum	1140.3	−23.9	0.0000	0.0000
PR-42	Prevotella_sp_HOT_317	288.0	−25.3	0.0012	0.0074
CA-17	Capnocytophaga_sp_HOT_332	223.8	−26.2	0.0016	0.0091
AC-43	Actinomyces_odontolyticus	87.5	−27.0	0.0016	0.0091
SR-01	SR1[G-1]_sp_HOT_345	321.9	−28.6	0.0003	0.0031
AC-22	Actinomyces_sp_HOT_178	100.7	−29.9	0.0076	0.0281
PR-15	Prevotella_micans	687.0	−33.9	0.0154	0.0473
LA-03	Lachnoanaerobaculum_sp_HOT_083	75.9	−40.1	0.0004	0.0031
GP-072	Pseudomonas_Genus_probe	113.8	−45.0	0.0053	0.0221
CA-03	Campylobacter_curvus	71.9	−48.4	0.0032	0.0159
LE-12	Leptotrichia_sp_HOT_219	530.0	−49.4	0.0001	0.0009
LA-29	Lautropia_mirabilis	8113.6	−55.9	0.0000	0.0000
PR-71	Prevotella_saccharolytica	214.3	−60.6	0.0003	70.0031
CO-03	Corynebacterium_matruchotii	3137.4	−61.6	0.0000	0.0000
PR-72	Prevotella_saccharolytica	169.6	−71.0	0.0011	0.0070
TA-02	Tannerella_sp_HOT_286	1137.6	−91.4	0.0000	0.0000
PO-16	Porphyromonas_catoniae	329.7	−91.9	0.0005	0.0043
BA-03	Bacteroidales[G-2]_sp_HOT_274	1761.5	−99.3	0.0000	0.0006
AC-10	Actinomyces_johnsonii	67.7	−100.5	0.0001	0.0010
LA-31	Lachnospiraceae[G-3]_sp_HOT_100	527.2	−102.8	0.0000	0.0008
GP-022	Cardiobacterium_Genus_probe	1195.8	−110.2	0.0000	0.0000
AG-03	Aggregatibacter_paraphrophilus	286.5	−112.5	0.0006	0.0046
GP-076	Selenomonas_&_Centipeda_Genus_probe	1541.7	−155.9	0.0000	0.0000
AC-11	Actinomyces_massiliensis	186.1	−201.7	0.0000	0.0000
LA-09	Lachnospiraceae[G-3]_sp_HOT_100	242.2	−215.3	0.0000	0.0000
AC-18	Actinomyces_sp_HOT_170	636.4	−389.8	0.0000	0.0000
TM-13	TM7[G-1]_sp_HOT_348	2241.3	−514.4	0.0000	0.0000

Microorganisms exhibiting positive value fold changes (shaded in gray) indicate higher prevalence in caries-active children compared to caries-free children. Microorganisms exhibiting negative value fold changes (unshaded) indicate higher prevalence in caries-free children compared to caries-active children.

### Significant sex-specific differences in salivary microbiota in caries-active and caries-free children

*Moraxella* genus probe 2, *Streptococcus* genus probe 2, *N. flavescens, Rothia aeria*, and *H. pittmaniae* were found at significantly higher levels in caries-active boys than caries-active girls, exhibiting 171-, 26.4-, 20.7-, 3.9- and 3.8-fold differences, respectively (Table 2). Alternately, *Lactococcus lactis, Selenomonas* species HOT 126, *Actinobaculum* species HOT 183, *Lachnoanaerobaculum* species HOT 083, *Prevotella* species HOT 315, *Veillonella parvula*, and *Alloprevotella* species HOT 473 were found at significantly higher levels in caries-active girls than caries-active boys, exhibiting 92.2-, 21.5-, 9.8-, 8.9-, 7.3-, 5.4- and 4.4-fold differences, respectively (Table 2).

Conversely, for caries-free children, *Lachnospiraceae* [G-3] species HOT 100 and *Bergeyella* species HOT 900 were found in significantly higher levels in caries-free boys than caries-free girls, exhibiting 39.5 and 21.8-fold differences, respectively (Table 3). In addition, *Selenomonas noxia, Prevotella micans, Prevotella oralis, Actinomyces* species HOT 170, *Aggregatibacter* species HOT 949, *Leptotrichia* species HOT 218, *Oribacterium* species HOT 078, *Leptotrichia* species HOT 498, *Actinomyces* species HOT 171, *Streptococcus anginosos, Leptotrichia wadei, Prevotella pleuritidis, Cardiobacterium* genus probe, *Selenomonas sputigena*, and *Actinomyces naeslundii* were found at significantly higher levels in caries-free girls than caries-free boys, exhibiting 357-, 299-, 206-, 159-, 137-, 102-, 87.3-, 78.7-, 69.4-, 49.4-, 37.4-, 28.7-, 28.6-, 26.8-, and 16.9-fold differences, respectively (Table 3).

Microorganisms were also compared between caries-active children as a combined cohort compared to microorganisms contained within caries-free children (Table 4). Fold changes were also determined for each microorganism contained within caries-active children versus caries-free children and then ranked from high positive values (indicating a high prevalence of specific microorganisms within caries-active children) and extremely low negative values (indicating a high prevalence of specific microorganisms within caries-free children). Microorganisms displaying statistically significant fold changes (> 1.5 fold changes with FDR adjusted p < 0.05) are displayed in Table 4; gray shading indicates a high prevalence of microorganisms found in caries-active children compared to caries-free children. This table lists only those microorganisms displaying significant fold changes according to caries status, and omits the large majority of species (hundreds) that exhibit nominal or minor fold changes between caries-active children and caries-free children.

## Discussion

### Salivary microbiota and influence on oral and systemic health and disease

The salivary microbiota has direct and significant implications on oral and systemic health, and may influence the development of dental caries and other dental diseases, including endodontic infections and periodontal disease. Salivary microorganisms commonly found to be associated with caries include *Atopobium, Streptococcus*, and *Veillonella* [23–25]. Other salivary microorganisms associated with caries also include members from *Actinobaculum, Aggregatibacter, Rothia, Granulicatella, Gemella, Actinomyces, Selenomonas, Haemophilus, Megaspora, Prevotella, Bacteroides*, and *Bifidobacterium* [23–27]. Caries-associated bacteria within the *Prevotella* genus include *Prevotella denticola* and *Prevotella histicola* [28,29]. Conversely, a number of salivary microorganisms are associated with caries-free status, including *Porphyromonas pasteri, Streptococcus* Genus Probe 4, *Bergeyella* species HOT 322, and *Corynebacterium durum* [23,30,31]. Microbiota identified within caries-free individuals also include *Actinomyces, Bergeyella, Campylobacter, Granulicatella, Kingella, Leptotrichia, Shuttleworthia satelles, Rothia, Treponema, Peptococcus, Porphyromonas catoniae*, and *N. flavescens* [23,25,30,32]. Microorganisms associated with endodontic infections include *P. denticola, Granulicatella adiacens*, and *Capnocytophaga sputigena* [28,33–36].

Numerous members of the salivary microbiome are associated with periodontitis in varying levels of severity. *SR1* [G-1] species HOT 345 and *P. denticola* and *Prevotella* species HOT 317 were found to be associated with periodontitis or serve as risk indicators for periodontitis [37–39]. *TM7* Genus Probe was associated with generalized gingivitis [40]. Microorganisms implicated in refractory periodontitis include *G. adiacens, Gemella sanguinis*, and *S. noxia* [41–43]. Those microorganisms associated with generalized aggressive periodontitis include *G. adiacens, S. noxia, Bacteroidales* [G-2] species HOT 274, and *Cardiobacterium* Genus Probe [37,40,41,44,45]. *S. noxia* and *Bacteroidales* [G-2] species HOT 274 are also associated with chronic periodontitis [45,46]. Conversely, *C. sputigena* was associated with periodontal health [43,47].

### Rationale for examining the *s*alivary microbiome and influence of saliva on the oral microbiome and dental caries

The oral microbiome encompasses heterogeneous microorganisms from different sites of the mouth, including periodontal sulcus, dental plaque, tongue, buccal mucosa and saliva (48). Saliva contains little if any indigenous microbiota, instead acquiring bacteria shed from desquamating oral epithelial surfaces and oral biofilms, including dental plaque. Dental plaque contains *Streptococcus mutans*, one significant microbial contributor to dental caries and tooth decay, as well as other adherent microorganisms. Therefore, the salivary microbiome is considered to be an important reservoir that contains microorganisms found throughout the oral cavity [48,49], and upon interaction with the gut microbiome, may influence the onset of systemic diseases including inflammatory bowel disease. Thus, defining the factors underlying the constituency of the salivary microbiome are critical in understanding the complete oral microbiome and its influence in oral and systemic health and disease.

### Sex differences in the salivary microbiome of caries-active and caries-free children

Our data demonstrate significant differences in the salivary microbiome between caries-active and caries-free children, consistent with the known shifts in acidity of the oral cavity in the development of dental caries. For caries-active boys, *Streptococci* may represent more important determinants in the onset of caries, while for caries-active girls, *Veillonella* may be more important factors. The high prevalence of *Actinomyces*, of which some species are known to have cariogenic potential, in caries-free girls (Table 3), indicates that this microorganism may not be a major influential determinant in dental caries. The detection of several periodontal microorganisms, including *S. sputigena, S. noxia*, and *Aggregatibacter* species HOT 949, in caries-free girls, is consistent with the more neutral pH found in the non-cariogenic oral cavity. In caries-active girls, *Alloprevotella* species HOT 473 was the only species that exhibited significant sex differences and moderate abundance within the total microbial population (Tables 1 and 2).

### Other non-microbial factors influencing sex differences in dental caries

Shaffer et al. [1] have indicated that genetics may significantly influence variability in dental caries experience between individuals. In genome-wide scans, the genetic region signifying the potential for ‘higher caries experience’ was located at 14q24.3, which is in close proximity to ESRRB (estrogen-related receptor beta) [16]. ESRRB is similar to the estrogen receptor and contributes to various biological functions in physiology and pathology [50]. High expression of ESRRB in females may lead to higher caries rates due to increased enamel surface sensitivity towards demineralization in the acidic oral environment [51].

Genes expressed in enamel mineralization, *AMELX (amelogenin)* and *AMNB*, as well as *ESRRB* were found to be associated with calcium levels in saliva. Genetic mutations in enamel mineralization are directly responsible for X-linked *Amelogenesis Imperfecta*, which supports their contribution in enamel matrix formation and caries vulnerability [52]. Deeley et al. [53] found that ‘higher caries experience’ was associated with irregular *amelogenin* allele markers. This may occur in the presence of X-inactivation and mosaicism and may contribute to female caries susceptibility [53].

Depending on the type and frequency of sugar introduced in the diet, a cariogenic oral environment may also develop via fermentation of sugars by acidogenic microorganisms, resulting in enamel decalcification [12]. Vegetarian diets, which contain sugar-containing fruits and berries, have been shown to increase caries incidence [54,55]. Higher caries prevalence was found in participants with vegetarian diets, while protein-meat consumption was suggested to be caries-protective because of the development of a more alkaline oral environment due to protein metabolism [56]. Females were noted to engage more in vegetarianism than males [57], with western societies containing more vegetarian women than vegetarian men, and women consuming substantially less meat in comparison to men [54].

Throughout the female lifespan, various biological stages are influenced by reproductive hormone fluctuations that coincide with changes in body structure. Estrogen fluctuations that occur during pregnancy influence suppression of the immune system and have known impact on salivary composition and flow rate contributing to a less protective oral environment and greater caries susceptibility. The parotid, buccal and labial salivary glands demonstrated lower flow rates in females than males, with elderly participants demonstrating substantial differences [15,58]. The minor salivary gland in women consistently contained substantially lower IgA concentrations among all tested salivary glands, and the higher IgA concentration found in men may provide greater protective anti-cariogenic effects in the oral cavity when compared to women [15].

Oral health disparities in men and women can be influenced by cultural and social differences [59]. Overall, dental caries unequally affects ethnic minorities and low socioeconomic status populations, with women in these categories being more affected than men [60]. Female-inclined behaviors, such as labor and domestic role in food preparation, may contribute to increased caries rates known to be associated with women. This role provides easy access to food supplies and a greater tendency towards frequent snacking [61]. Some countries exhibit this trend in caries incidence due to social and religious involvement, including son preference, fasting, and pregnancy-related dietary restrictions [62].

### Limitations of the study and concluding remarks

One limitation of the study concerns the difficulty in obtaining caries-free children, which was primarily due to the referral-based, region-wide patient acquisition system at the OHSU School of Dentistry. This referral system resulted in the predominant availability of more difficult and problematic dental cases, including advanced caries, and very few caries-free individuals. We supplemented the caries-free cohort with additional patients obtained from an external practitioner’s clinic, but we do acknowledge that increasing the caries-free control population may increase the statistical power of the study, and would have the potential to further discriminate borderline differences in microbial species between the sexes. Another limitation was the use of participants in different developmental dental stages – individuals with primary dentition or mixed dentition. We initially started the study attempting to identify only young individuals – ages 2–6 – with only primary dentition, but were not able to identify sufficient numbers of participants, and later expanded our age range to age 12 years. The majority of participants in this study were ages 2–6 years (30 boys and 34 girls) and the smaller remainder (11 boys and 13 girls) were within the 7–12 years age range, many with mixed dentition and eruption of permanent central and lateral incisors, first molars and cuspids. The limitations of different developmental stages were best mitigated with the use of saliva specimens instead of plaque specimens from specific teeth, many of which were missing or replaced with permanent teeth. The use of saliva also facilitated investigation of the salivary microbiome, perhaps the most reflective of the entire repertoire of microorganisms contained within the oral cavity. Any potential limitations were also mitigated with the use of our advanced multi-stage statistical analyses, which use the criterion of abundance and statistical differences in fold changes to identify the most relevant microbial species based on caries-status and by sex.

As the primary finding in our studies, in caries-active boys, *N. flavescens* and *H. pittmaniae* were the only two microorganisms (shaded in gray in Table 1) that exhibited both significant sex differences (> 1.5-fold changes with FDR adjusted p < 0.05) and abundance comprising > 0.5% of the microbial population (Tables 1 and 2). In caries-active girls, *Alloprevotella* species HOT 473 was the only species that exhibited both significant sex differences (4.4-fold difference between caries-active girls and caries-active boys; p = 0.0003) as well as high abundance in numbers (1.85% of the total microbial population). *Alloprevotella* species are associated with neutral pH in the oral cavity and has been found in cases of caries progression, while *Neisseria* species are considered to be acid producers and are more conducive to the presence within a cariogenic environment. Determining the causative reason why *Alloprevotella* species are found more predominantly in caries-active girls, or conversely why *N. flavescens* and *H. pittmaniae* are found more predominantly in caries-active boys, is beyond the scope of this study, and could be potentially dictated by genetic, nutritional, hormonal or immune factors or mechanisms, all of which could represent avenues for future investigation.
